# Crystal phase engineering of silicene by Sn-modified Ag(111)†

**DOI:** 10.1039/d3nr01581e

**Published:** 2023-05-02

**Authors:** Simona Achilli, Daya Sagar Dhungana, Federico Orlando, Carlo Grazianetti, Christian Martella, Alessandro Molle, Guido Fratesi

**Affiliations:** a ETSF and Physics Department “Aldo Pontremoli”, University of Milan via Celoria 16 Milano I-20133 Italy guido.fratesi@unimi.it; b INFN, Sezione di Milano I-20133 Milano Italy; c CNR-IMM Agrate Brianza Unit via C. Olivetti 2 Agrate Brianza I-20864 Italy alessandro.molle@mdm.imm.cnr.it

## Abstract

The synthesis of silicene by direct growth on silver is characterized by the formation of multiple phases and domains, posing severe constraints on the spatial charge conduction towards a technological transfer of silicene to electronic transport devices. Here we engineer the silicene/silver interface by two schemes, namely, either through decoration by Sn atoms, forming an Ag_2_Sn surface alloy, or by buffering the interface with a stanene layer. Whereas in both cases Raman spectra confirm the typical features as expected from silicene, by electron diffraction we observe that a very well-ordered single-phase 4 × 4 monolayer silicene is stabilized by the decorated surface, while the buffered interface exhibits a sharp 
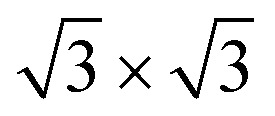
 phase at all silicon coverages. Both interfaces also stabilize the ordered growth of a 
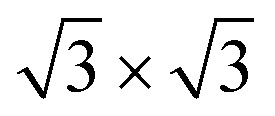
 phase in the multilayer range, featuring a single rotational domain. Theoretical *ab initio* models are used to investigate low-buckled silicene phases (4 × 4 and a competing 
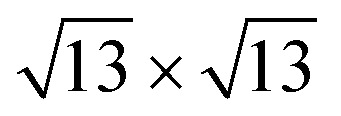
 one) and various 
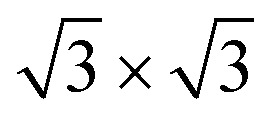
 structures, supporting the experimental findings. This study provides new and promising technology routes to manipulate the silicene structure by controlled phase selection and single-crystal silicene growth on a wafer-scale.

## Abstract 2

Engineering the silicene/Ag(111) interface by tin decoration or buffering by a stanene layer allows us to grow single-phase silicene both at monolayer and multilayer thickness, as revealed by diffraction/Raman and *ab initio* studies.

## Graphical abstract

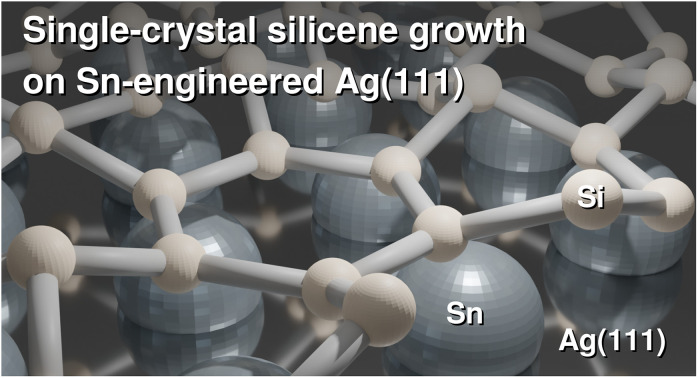

## 1 Introduction

Monoelemental two-dimensional (2D) materials named Xenes^1–3^ have recently expanded the wealth of available 2D systems. Among these, silicene is the oldest graphene's cousin and has opened the way to a variety of potential applications ranging from nanoelectronics,^2,4,5^ optoelectronics,^6^ Li storage in batteries,^4,7^ thermoelectricity^8^ and biomedicine.^4,9^

Silicene can be grown by molecular beam epitaxy (MBE) on a suitable substrate: the most adopted one, ten years after the first realization, is probably still Ag(111). To date, it is well settled in the literature that the first monolayer (ML) of silicene on Ag(111) appears as the mixture of 4 × 4, 
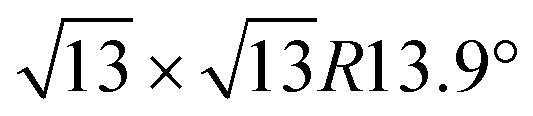
 type I and II, 
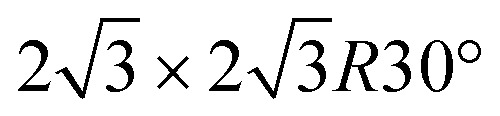
 and other minor reconstructions as a function of growth temperature.^10–12^ Likewise, once coverage reaches the second layer, multiple rotational domains of 
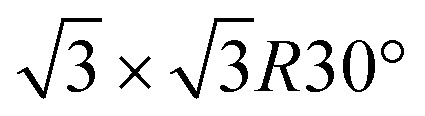
 are found.^13^ This implies the formation of polycristalline samples, where grain boundaries provide scattering centers for the charge carriers.^5^ Surface dewetting may even occur.^14^ The synthesis of large-scale mono- and multilayer misoriented-free single-crystals of silicene thus provides a great technological challenge, shared with more mature 2D materials, *e.g.*, graphene and hexagonal boron nitride.^15–17^ Concomitantly, decoupling silicene from the native Ag(111) surface is necessary to preserve its peculiar properties, otherwise lost in the formation of interface states.^18^ Suitable substrate modifications may tackle both issues. The use of a buffer layer interposed between the material to be grown and the substrate could be a viable way to synthesize more regular adlayers, as testified by a number of studies implementing graphene for this purpose.^19,20^ In this respect, stacking 2D heterostructures, as it would result by piling up other 2D materials together with silicene, is also an appealing way to tailor the properties of the final system,^21^ and stacking of Xenes have been indeed recently proposed.^22^ We have successfully demonstrated the feasibility of a heterostructure consisting of silicene and stanene layers, both grown on Ag(111).^23^ The stanene layer weakens the interaction between silicene and the silver substrate, allowing the silicene layers to respond to the thermal excitation as a quasi-free-standing layer.^24^ Remarkably, the early stage of tin evaporation over silver leads to the formation of an ordered Ag_2_Sn alloy in the first surface layer,^25^ that acts as a template for the growth of stanene.^26^ This suggests adopting the same surface alloy for the growth of silicene.

In this work we show how single-crystal silicene adlayers can be grown by MBE on the Ag(111) surface upon engineering the substrate, following two routes: by decorating it with tin atoms, hence forming Ag_2_Sn–Ag(111), and by predepositing a stanene buffer layer.

## 2 Experimental and computational details

The samples were grown using a MBE system equipped with *in situ* low energy electron diffraction (LEED) and Auger Electron Spectroscopy (AES). Three templates were considered: Ag(111), Ag_2_Sn–Ag(111) and Sn–Ag(111). The Ag(111) surface was prepared by cycles of Ar^+^ (1 kV/10 mA) at room temperature followed by annealing at 550 °C, each for 15 minutes. The two Sn-engineered Ag(111) surfaces are realized by depositing tin on pristine Ag(111). In the case of Sn–Ag(111), a complete coverage of ML stanene was ensured, whereas Ag_2_Sn–Ag(111) was obtained limiting the tin coverage to 1/3 ML.^26^ Silicon was deposited within the substrate temperature range of 200–225 °C at a rate of 0.04 ML per second. AES analysis carried out on mono- and multilayer silicene (reported in the ESI, Fig. S8†) is consistent with the silicon overgrowth with thickness. Finally, before unloading the samples from the MBE system, the samples were capped with amorphous and non-reactive Al_2_ O_3_ films (≈5 nm).^27^*Ex situ* Raman characterization was performed using a spectrometer equipped with a 514 nm (2.41 eV) solid state laser and a 50 × (0.75 numerical aperture) objective. The incident laser power during acquisition was kept below 1 mW to avoid sample damages. Sample homogeneity over the sample (1 × 0.5 cm^2^) was checked by repeating LEED and Raman spectroscopy measurements over different spots covering the whole surface area, ensuring the same diffraction and spectral features are consistently observed throughout the samples reported here.

Theoretical calculations were performed using Density Functional Theory (DFT) with the semilocal Perdew–Burke–Ernzerhof (PBE) gradient exchange functional^28^ and Grimme dispersion forces.^29^ We adopt pseudopotentials and a double-zeta polarized (DZP) atomic orbital basis set as implemented in the SIESTA code.^30^ We include in the calculation three layers of substrate, leaving free to relax the outermost one and the overlayer, and 20 Å of vacuum between periodic replicas.

## 3 Results and discussion

**Fig. 1:**
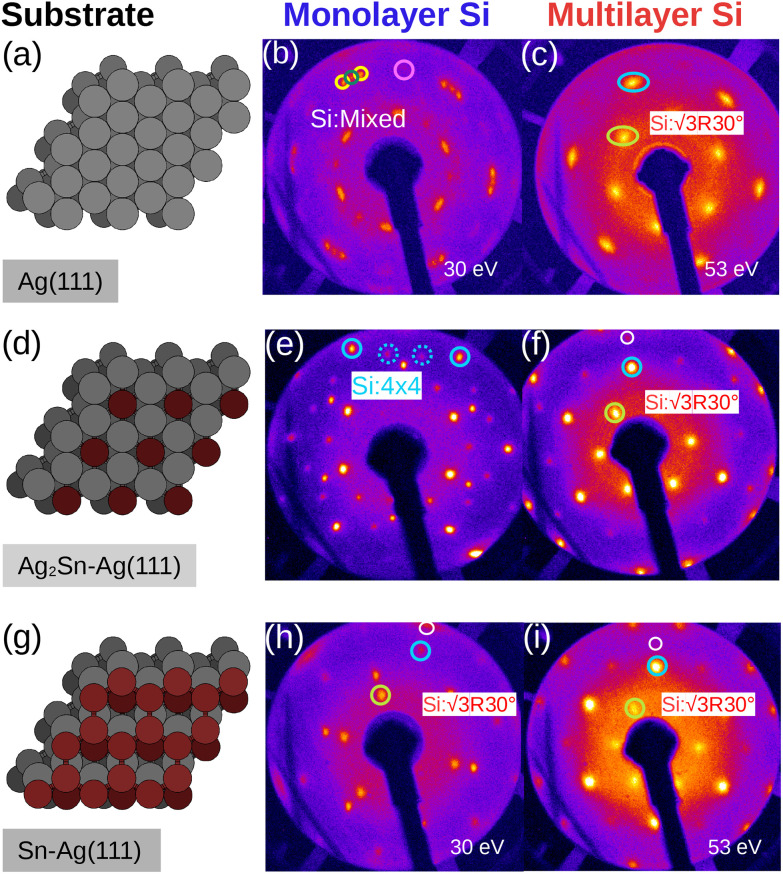
LEED patterns obtained after depositing silicene with varying thickness on different surface template. (a, d and g) are ballstick models for the surface templates, corresponding to the bare Ag(111), Sn-decorated Ag(111) and Sn-buffered Ag(111), respectively. (b, e and h) and (c, f and i) correspond to mono- and multilayer (≈3 ML) silicene synthesized on the three templates, respectively. In (b) the solid green circle is the integer order spot of the 4 × 4 silicene phase, the two yellow circles are 
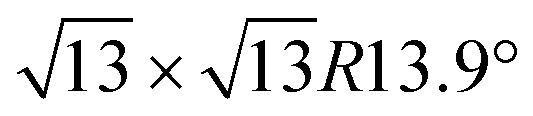
 type-I spots and the pink circle is 
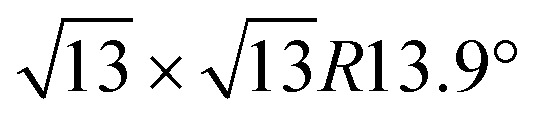
 type-II spot. In (c, e, f, h and i): cyan is silicon integer order (silicene-3 × 3/Ag(111)-4 × 4) and yellow is silicene- 
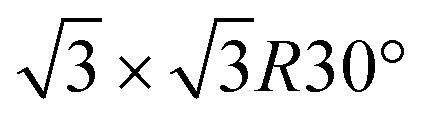
 spot or Ag(111)- 
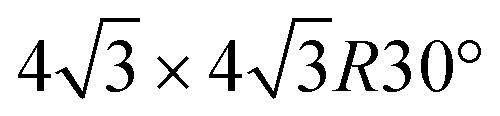
. Where shown, the white circle is Ag(111)-1 × 1.

**Fig. 2:**
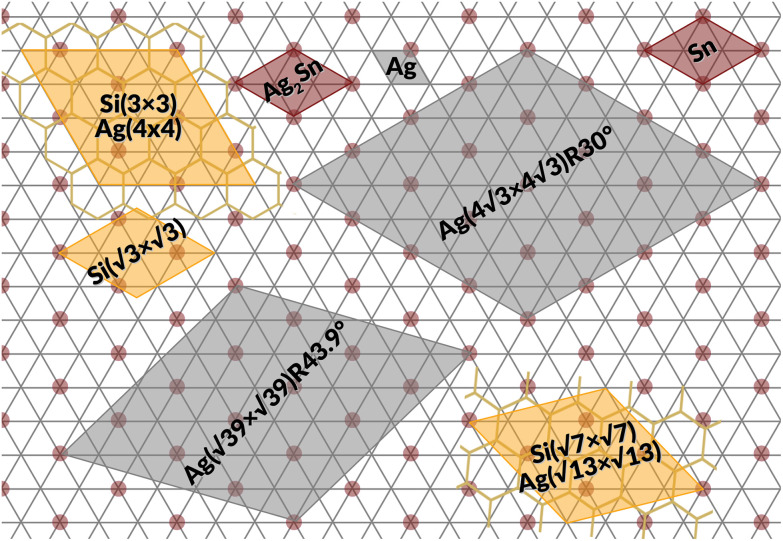
Ag(111) surface lattice (gray lines) showing tin atoms of Ag_2_Sn (brown circles), unit cells of Ag(111) (small gray area), Ag_2_Sn–Ag(111) and Sn–Ag(111) (brown areas), silicon structures (yellow areas), and Ag supercells used in the calculations (large gray areas).

Fig. 1 summarizes the LEED patterns acquired after depositing mono- (panels b, e, h) and multilayer silicene (3 MLs) (panels c, f, i) on the three substrates considered: from top to bottom, Ag(111), Ag_2_Sn–Ag(111) and Sn–Ag(111) (panels a, d and g). Fig. 2 shows real-space unit cells. Further analysis on the specific cases is given in the ESI.†

The case of silicene grown directly on Ag(111) serves as a reference. As shown in Fig. 1(b), the ML silicene is marked with silicene superstructures that show mixed phases:^31^ 4 × 4 (LEED spots circled in green), 
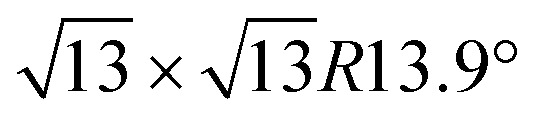
 type I (in yellow) and type II (in pink).

What is most alluring is that silicene superstructures on Sn-engineered surface templates, whether mono- (Fig. 1e–h) or multilayer (Fig. 1f–i), are free of mixed phases. On the Ag_2_Sn–Ag(111) and Sn–Ag(111) surfaces, the mono- and multilayer silicene deposited above do not hint any signs of multiple rotational domains at the macroscopic level that can be confirmed with the LEED spots which are sharp and well centered. Considering the ML case, it is interesting to note that well resolved LEED patterns in Fig. 1(e) as highlighted with cyan circles are outstandingly related to single phase 4 × 4 silicene on Ag_2_Sn–Ag(111) surface. This finding sharply discriminates the single-phase silicene by tin decoration (see Fig. 1(e)) from the directly grown mixed-phase silicene (in Fig. 1(b)) in the respective structural fashions. This is elaborated further in Fig. S2† where we also probe the growth on the same template starting from the submonolayer regime. Single-phase silicene is also found when ML silicene is grown on Sn–Ag(111) (in Fig. 1(h)) showing a 
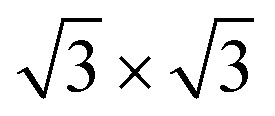
 crystal phase of silicene (see yellow circled LEED spots), in addition to the Ag- 
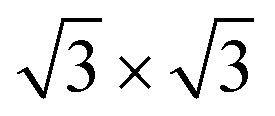
 spots as resulting from the Sn-engineered layer. We previously attributed this crystal phase of silicene to the 4 × 4 structure.^23^ Indeed, the cyan circles mark spots corresponding to a 4/3 real-space periodicity of silicon over Ag(111), as expected from the silicene-3 × 3/Ag(111)-4 × 4, and the green circles could be attributed to silicene patches locally exceeding the ML coverage and featuring a 
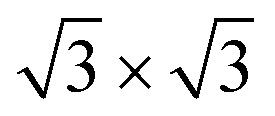
 reconstruction. This is however incompatible with our additional experiments, performed at 0.5 ML of silicon, that still produce the same LEED pattern with silicene- 
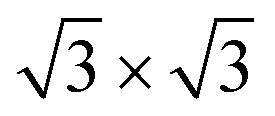
 peaks in Fig. S3.† Hence the same diffraction features persist from the submonolayer to the full layer and beyond (Fig. 1h), consistently pointing to a silicene- 
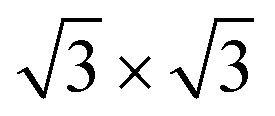
 phase already at low coverages. Also, we notice that fractional 4 × 4 spots visible for silicene/Ag_2_Sn–Ag(111) (dotted circles in Fig. 1e) are not visible on Sn–Ag(111). Also see Fig. S7† for simulated LEED patterns. Overall, this picture allows us to reinterpret the structure obtained at ML Si/Sn–Ag(111) as a 
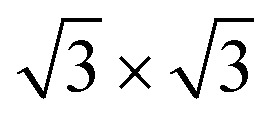
 phase. These findings suggest that a carefully tailored tin atomic or sub-atomic coverage on the Ag(111) surface has a special role in determining whether ML silicene is 4 × 4 or 
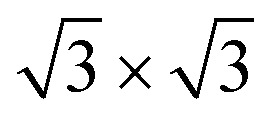
 as found at 1/3 ML tin coverage and on stanene, in both cases yielding a single-crystal silicene phase.

Considering the multilayer growth regime, Si- 
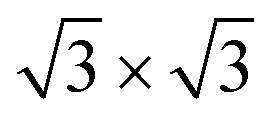
 is observed in all the cases, confirming that this phase is the energetically stable silicene surface (Fig. 1c, f and i). However, a remarkable difference emerges comparing multilayer silicene grown directly on Ag(111), that exhibits rotational domains^13^ (further elaborated in the ESI, Fig. S6†), whereas on both Sn-engineered substrates a single domain at 30° from the Ag(111) azimuths appears. This may be a result of the single-phase obtained for the first silicene layer growth on Sn-engineered Ag(111) substrates.

**Fig. 3:**
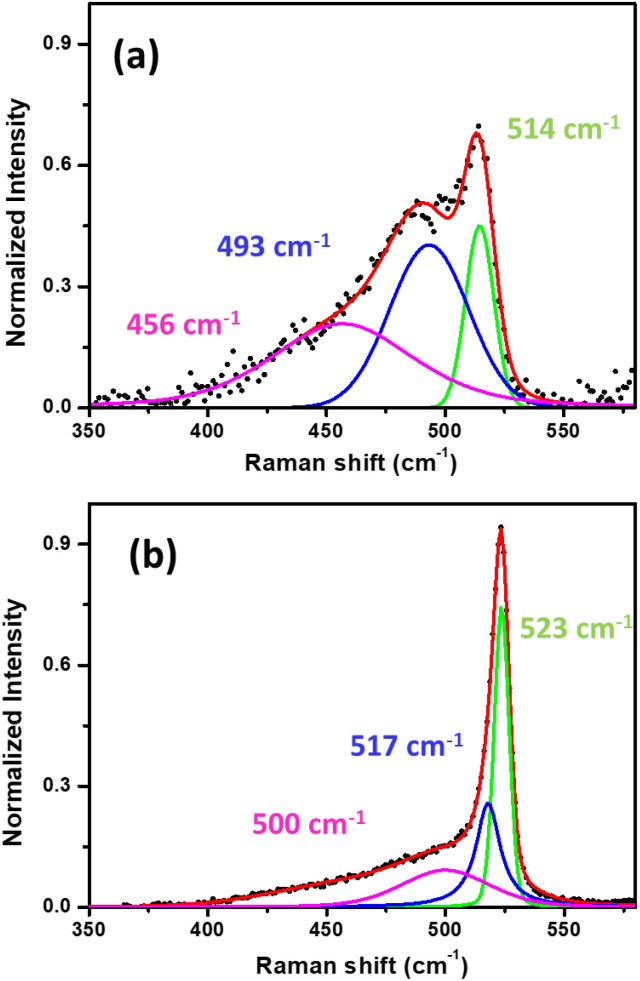
Raman spectra obtained from: (a) 1 ML and (b) 6 ML deposited on Ag_2_Sn–Ag(111) template. The black-dotted curves are the as acquired raw spectra, red curves are the fitted spectra where the green, blue and purple are three fitted Lorentzian–Gaussian components following the background subtraction.

In Fig. 3, we present the *ex situ* Raman spectra acquired from mono- (Fig. 3(a)) and multilayer silicene (Fig. 3(b)) grown on Ag_2_Sn–Ag(111) template. As previously reported for the single layer^32^ and multilayer,^33,34^ the shape profile of the Raman spectra exhibit a major peak and an asymmetric shoulder that are consistent with an in-plane and an out-of-plane breathing mode, respectively.^35^ In silicene on Ag(111) and Sn–Ag(111), the spectra are characterized by some fingerprints that allow for a quick identification of silicene in the *ex situ* environment.^23,25,35,36^ The decomposition of these Raman spectra into more than one component (see green, blue and purple Lorentzian-Gaussian components) confirms their asymmetric shape and is consistent with the typical silicene-on-Ag(111) and silicene-on-stanene-Ag(111) fingerprints.^23,35^ This agrees with the crystalline-Si phonon modes (less distribution in inter-atomic bond length) in the case of ML sample (Fig. 3(a)). The main silicene peak (green curve) is centered at a Raman shift of 514.2 and 523.4 cm^−1^ for mono- and multilayer silicene, respectively. These findings are in agreement with previous reports where the ML silicene is qualified by highly tensile strain (<520.5 cm^−1^),^35^ whereas a compressive strain takes place in the multilayer configuration with the main Raman peak shift being >520.5 cm^−1^.^34^ In the case of ML silicene, the primary peak refers to the E_2g_ mode of the freestanding silicene, whereas the secondary, less intense peaks are due to the out-of-plane breathing mode.^35^ Similarly, the blue-shift in the spectrum of the multilayer silicene enunciates the associated mixed sp^2^–sp^3^ hybridization, making it different from bulk crystalline silicon.^34^ In spite of the structural diversity, the mono- and multilayer silicene grown by Sn-buffering display a similar behavior with respect to those obtained by Sn decoration as previously reported.^23,24^

We have performed a thorough theoretical analysis of five different silicene-reconstruction/silver-superstructure combinations: the silicene-3 × 3/Ag(111)-4 × 4, the silicene 
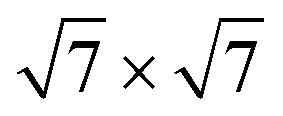
 /Ag(111)- 
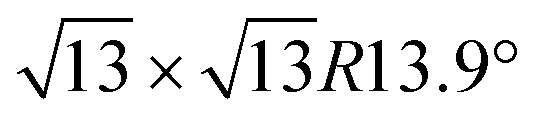
 (4 × 4 and 
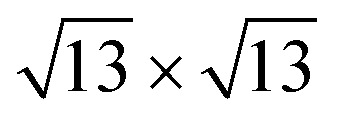
 in the following, respectively) [*i.e.*, the main monolayer periodicities we observe on Ag(111)] and three for silicene- 
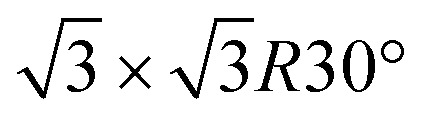
 /Ag(111)- 
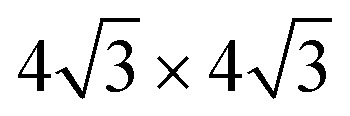
 (
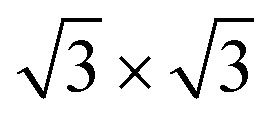
 in the following), namely, trigonal dumbbell silicene (TDS) and honeycomb dumbbell silicene (HDS)^37^ as well as Si trimers (SiT),^38^ with nominal silicon coverage of 1.16, 1.33, and 1.5 ML, respectively. To match the periodicity of Sn-modified Ag(111)- 
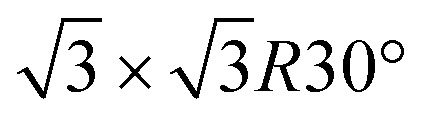
 we adopt surface supercells depicted in Fig. 2. In particular, a Ag(111)- 
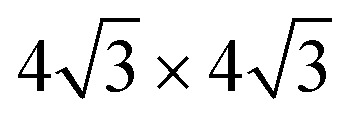
 supercell for both the 4 × 4 and the 
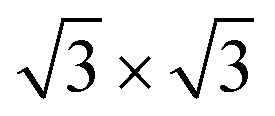
 phases and a Ag(111)- 
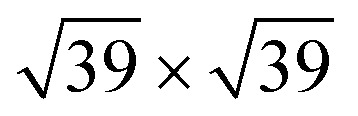
 supercell for the 
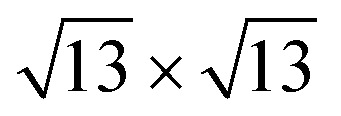
 phase. The resulting 15 combinations have been structurally optimized. Selected cases are presented below (see Fig. S9† for all combinations).

**Fig. 4:**
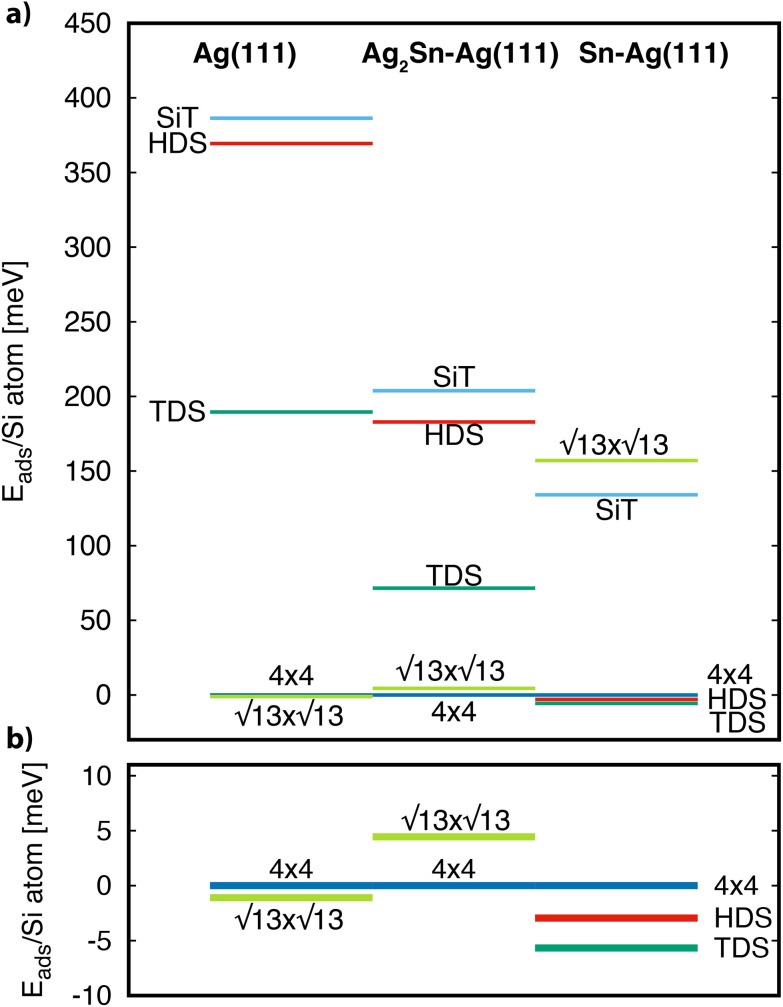
(a) Adsorption energy per Si atom of different silicene reconstructions on the Ag(111), Ag_2_Sn–Ag(111) and Sn–Ag(111) substrates. Values are referred to the 4 × 4 case. (b) Enlargement of the most stable cases.

The calculated adsorption energy per Si atom, referred to the value of the 4 × 4, are reported in Fig. 4. On both Ag(111) and Ag_2_Sn–Ag(111) the 4 × 4 and 
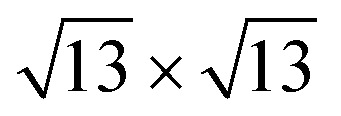
 are the most stable superstructures, whereas the phases with 
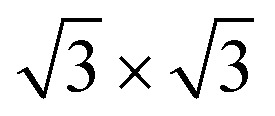
 symmetry lie at higher energies (≈70 and ≈400 meV). This result supports the experimental finding relative to the absence of 
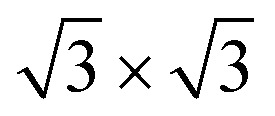
 phases on these two substrates for <1 ML silicene. We also observe, although changes are very subtle, that the energy difference between the 4 × 4 and the 
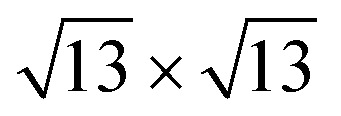
 favors the 4 × 4 as we move from the bare Ag(111) to Ag_2_Sn–Ag(111), in agreement with the experimentally observed stabilization of 4 × 4 silicene. Moving to the Sn–Ag(111) surface 
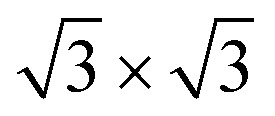
 TDS and HDS reconstructions eventually become more stable than the 4 × 4. Being the comparison about energies per atom (not per surface area) implies that stanene-buffered Ag(111) stabilizes the 
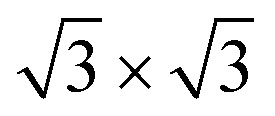
 phases already before ML silicene completion, in agreement with the LEED results below the ML (Fig. 1h, Fig. S3†). The 
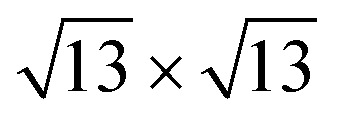
 is pushed higher in energy, above the SiT which is substantially unstable on all the substrates. The small energy differences among the most stable structures would not allow *per se* to rule out competing phases, but the general trends found from the calculations form a consistent picture together with the experimental findings of a dominant silicon morphology depending on the substrate engineering process.

**Fig. 5:**
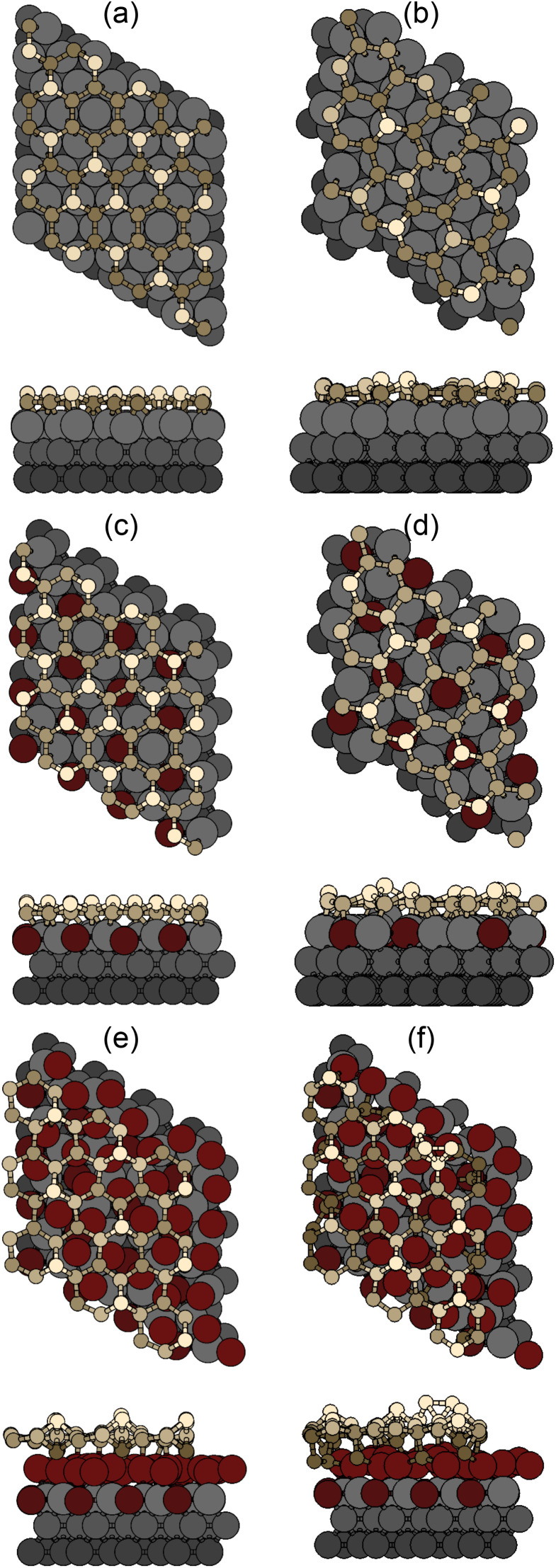
Top and side views of model structures for the most stable phases: (a) 4 × 4 and (b) 
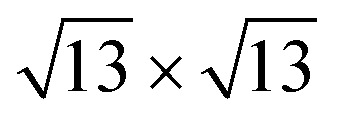
 on Ag(111); (c and d) the same on Ag_2_Sn–Ag(111); (e) TDS and (f) HDS 
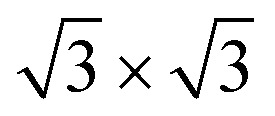
 on Sn–Ag(111). Si atoms are represented as yellow spheres, Sn as red and Ag as gray ones; darker colors are used for deeper atoms.

We now describe geometrically and electronically the properties of the most stable structures as seen in Fig. 4(b), namely, the 4 × 4 and 
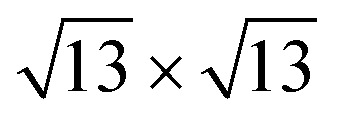
 on Ag(111) and Ag_2_Sn–Ag(111) substrates and the TDS and HDS 
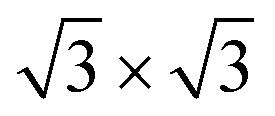
 cases for Sn–Ag(111). The optimized structures are reported in Fig. 5. On Ag(111), the 4 × 4 and 
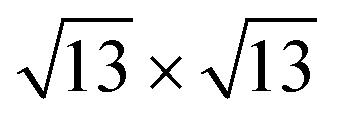
 reproduce the known structures.^39^ When the two are realized on Ag_2_Sn–Ag(111), we found that the overall geometry is mildly affected in the height of the Si atoms (see Table S1†), whereas the overall pattern of protruding atoms is mostly preserved: buckling in the Si layer increases by 0.12 Å in the 4 × 4 and by 0.40 Å in the 
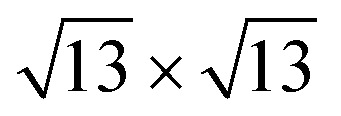
; in the latter, the most protruding Si atoms sit atop Ag atoms that are lifted out, accompanying their further height increase. The TDS and HDS structures are characterized by the presence of pairs of Si atoms standing one on top of the other (dumbbells)^37^ within a 
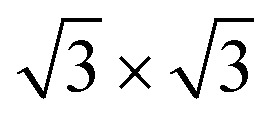
 reconstruction of silicene (see *e.g.*, the bright Si atoms in Fig. 5e). We find a Si–Si distance in the dumbbell of 2.56 Å in both cases on Ag(111), that increases by 0.2 (0.1) Å in TDS (HDS) on Sn–Ag(111). Remarkably, the structure of TDS is nearly unchanged whereas that of HDS presents an up/down arrangement of dumbbells, that are also incorporated within the Sn lattice. We point out that Sn–Si intermixing is not expected from the experimental core-level shifts;^23^ from our calculations, the intermixing we observe is mostly “geometrical”, as the analysis of charge transfer (below) shows minimal transfer between Si and Sn also in this case. For the 4 × 4/Sn–Ag(111) case we refer to our previous work.^23^

**Fig. 6:**
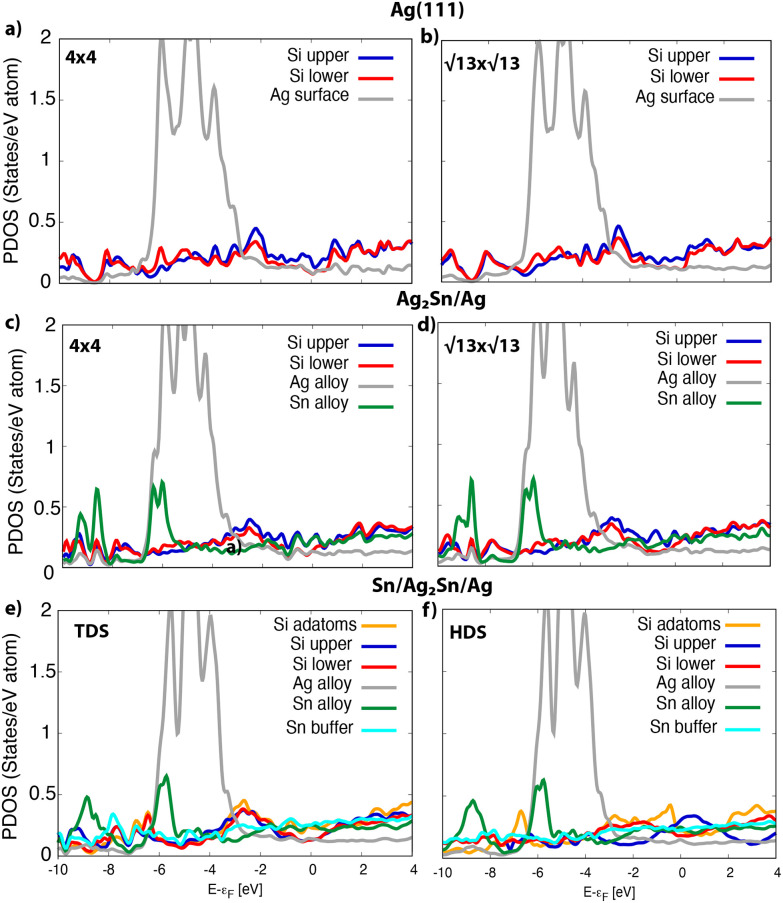
Density of states, resolved for chemical species, of the most stable phases on different substrates: (a) 4 × 4 and (b) 
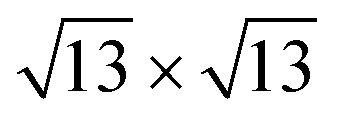
 on Ag(111); (c and d) the same on Ag_2_Sn-Ag(111); (e) TDS and (f) HDS on Sn-Ag(111).

In Fig. 6 the projected density of states (PDOS) on different species is reported for the systems in Fig. 5. For silicene on silver the PDOS on Si atoms features very similar structures in both the 4 × 4 and the 
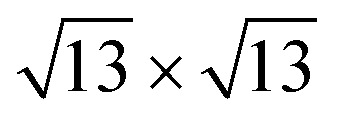
 (see Fig. 6a and b). The projection on the outermost Si atoms (blue) and the ones near the surface (red) evidences a partial intra-layer hybridization, while the interaction with the substrate (gray) appears in the coincidence of peaks at the Fermi level (see also Fig. S11a and d†). On Ag_2_Sn–Ag substrate, the coincidence of some peaks in the PDOS on the upper and lower silicon (blue and red lines, respectively) is preserved (see Fig. 6c and d). In addition, in the range [−2,2] eV, a superposition with features of the PDOS on Sn atoms of the alloy (green) is evident (Fig. 6c), being favoured by the p_*z*_ symmetry of these states, protruding outwards, toward tin (see Fig. S10†). Notably, the interaction with tin and silver in the alloy also involves atoms of the silicon upper layer (blue in Fig. 6c) positioned on top of substrate atoms, despite they are not in direct contact with them (Fig. 5c).

On the other hand, the Si–Sn hybridization in the 
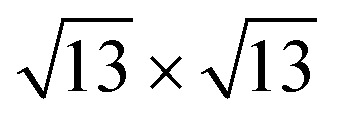
 /Ag_2_Sn–Ag(111) is milder (see Fig. 6d). The zoom of the PDOS around the Fermi level (Fig. S11d†) shows indeed the absence, in this case, of superimposed features belonging to Si, Sn and Ag, that are instead present for the other stable structures (Fig. S11a–c†). The reduced hybridization between p_*z*_ states of silicene with the ones of the substrate can eventually explain the lower stability of the 
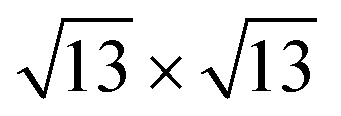
 /Ag_2_Sn–Ag(111) phase. This hypothesis is supported also by the comparison between the PDOS of TDS and HDS (Fig. 6e and f). In the PDOS of the most stable TDS there is an overlap between states in the silicon layer (orange, blue, red lines for adatoms, top and lower silicon, respectively) and the states of tin both in the stanene-like buffer (cyan) and in the alloy (green) (−7, −3, and 2 eV). Conversely, this coincidence of peaks is reduced in HDS which indeed turns to be less stable (Fig. 6f). A similar mild overlap between different species characterizes also the PDOS of the 4 × 4 and 
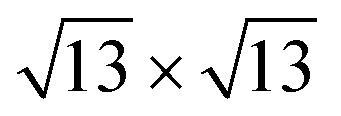
 on Sn–Ag(111) (Fig. S11e and f†).

**Fig. 7:**
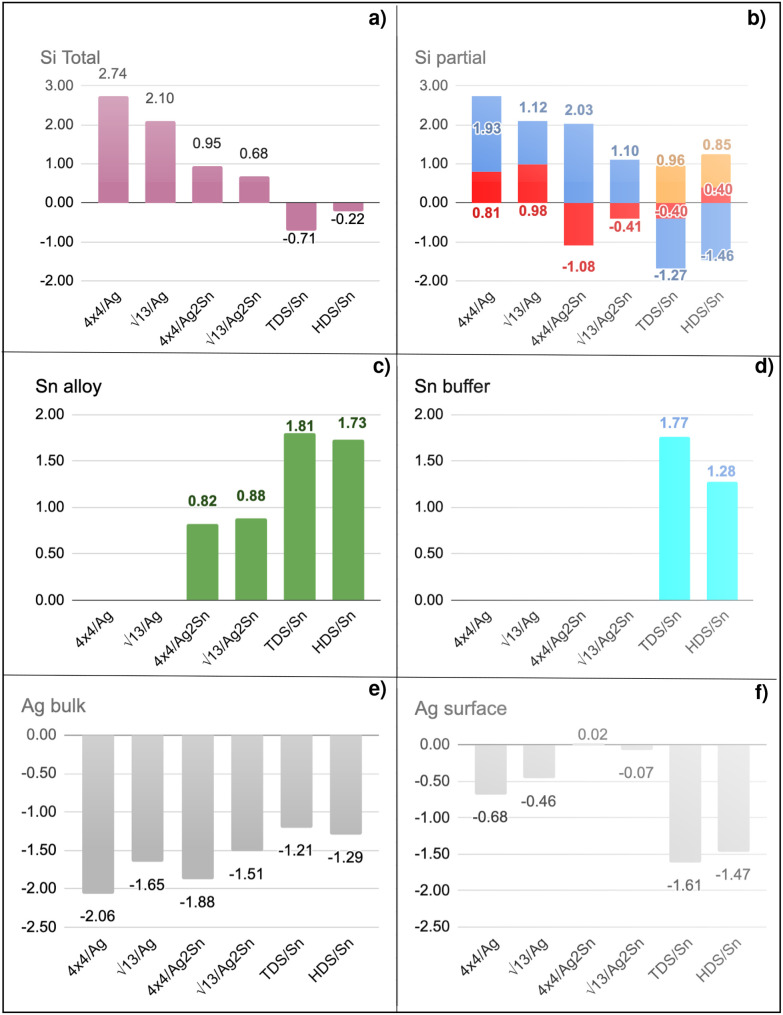
Charge transfer resolved for atomic species in selected configurations as indicated in the labels below the histograms. (a) Total charge transfer on Si atoms; (b) the same, separating upper (blue) and lower (red) silicon atoms in the overlayer and extra adatoms (orange) for the phases exceeding the monolayer; (c) on Sn atoms in the Ag_2_Sn layer; (d) on Sn atoms in the Sn buffer; (e) on subsurface and (f) surface Ag atoms.

The analysis of the Mülliken charges summarized in Fig. 7 evidences for the 4 × 4 and 
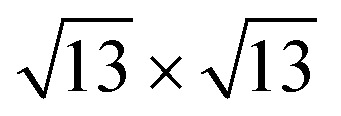
 on Ag(111) a charge transfer from the silver substrate to the silicon overlayer (Fig. 7a) that mainly involves the Si atoms in the upper layer (Fig. 7b), being the majority of these on top of silver surface atoms. Differently, for both the phases on Ag_2_Sn–Ag(111), the net charge transferred to silicon is lower and results from an electron increase on the upper silicon atoms (Fig. 7b, blue) and a depletion on the lowermost ones (Fig. 7b, red). This charge reduction can be eventually due to a charge redistribution in the Si overlayer and partially to the interaction with Sn atoms of the alloy, that collect charge (Fig. 7c). In the 
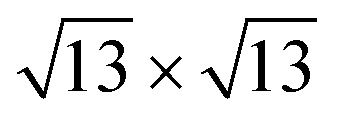
 on Ag_2_Sn–Ag(111) the net amount of charge transferred to silicene is ∼67% smaller than for the same phase on Ag(111) and ∼30% smaller than the 4 × 4 on the same substrate. Accordingly, the partial charge transfer on silicon sublayers is also smaller, being ∼50% of the one found for the 4 × 4 on Ag_2_Sn–Ag(111), confirming the reduced interaction between silicene and substrate. Finally, when silicene is adsorbed on Sn–Ag(111) the charge transfer is completely different from the previous cases, being the charge mainly collected by the buffer stanene layer (Fig. 7d) with both silicene and surface Ag atoms acting as donors. A sizeable charge transfer occurs also in the alloy plane, with charge depletion of Ag and accumulation on Sn atoms (Fig. 7c and f). Notably, the most stable TDS configuration is characterized by a quite large amount of electrons donated by the upper silicon layer and a smaller one by the lower silicon layer. On the contrary, the electronic charge is transferred to Si adatoms, suggesting intra-layer charge redistribution in silicene. The HDS configuration is instead characterized by a negligible charge transfer to silicene (Fig. 7a), even though an internal reorganization of electrons is still observed (Fig. 7b). It is worth noting that in HDS the lower Si atoms, despite sitting in the alloy plane, are not affected by a relevant charge transfer, suggesting that an out-of-plane interaction between alloy and silicene is preferred with respect to an intra-layer one in the plane of the alloy. Overall, this analysis confirms the different behaviour of the systems analyzed, showing a reduction of charge transfer in the less stable phases and complex charge redistribution depending on the local atomic structure.

## 4 Conclusions

We demonstrate with our *in situ* characterizations that decorating Ag(111) surface with Ag_2_Sn results in a suitable template to isolate a single-crystal silicene with 4 × 4 symmetry on a cm^2^ sample. This achievement is in line with the urgent and desirable request to provide wafer-scale high-quality 2D materials for electronic and optoelectronic applications. If a Sn buffer layer is piled on Ag_2_Sn–Ag(111) template, we find that 
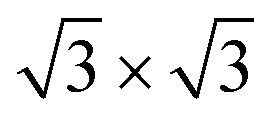
 silicene phase is stabilized even at low coverage, at variance with the bare Ag(111), where such a superstructure appears only in multilayer regime growth. Indeed, even on the Sn-engineered templates, the 
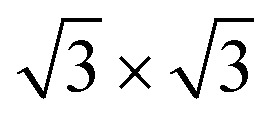
 silicene phase is common to all the multilayer motif irrespective of the surface template. However, most importantly, the multilayer silicene generated on Sn-engineered surface incorporates single-oriented domains with surface directions, at variance with the misalignment observed in the direct growth on pristine Ag(111) where rotational domains are present. We investigate theoretically the role of surface modifications by DFT models for low-buckled silicene phases (4 × 4 and 
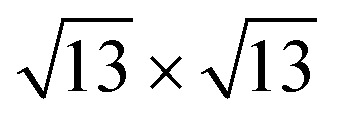
) and higher-coverage 
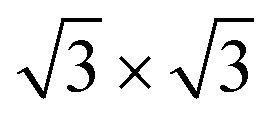
 structures as proposed in the literature. Calculated energies support the experimental findings: decorating the surface by tin atoms steers the energy difference between low-buckled silicene phases in favor of the 4 × 4 one; buffering with a tin layer makes dumbbell 
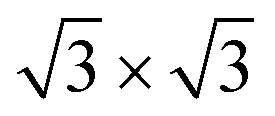
 phases become more stable than low-buckled ones already in the ML range. The presence of tin atoms at the interface is found to reduce the charge transfer between silver and silicene atoms.^15–17^ Silicene can be accommodated on diverse target substrates aiming at electronics and optoelectronics with large area manipulation of the silicene layer^40^ but multiphase or polycrystalline character of the pristine silicene hurdled so far a full control of its properties. Based on the current experimental and theoretical findings, we are now able to tailor a single-crystal self-organization of silicene *via* interface engineering. Our findings are preparatory for upgrading the silicene standard as an electronic material to be readily integrated in a device platform.

## Author contributions

S. A., conceptualization, formal analysis, investigation, validation, visualization, writing – original draft, writing – review & editing; D. S. D., conceptualization, investigation, validation, visualization, writing – original draft, writing – review & editing; F. O., formal analysis, investigation, validation, writing – review & editing; C. G., investigation, validation, writing – review & editing; C. M., investigation, validation, writing – review & editing; A. M., conceptualization, funding acquisition, writing – review & editing; G. F., conceptualization, formal analysis, investigation, validation, visualization, writing – original draft, writing – review & editing.

## Conflicts of interest

There are no conflicts to declare.

## Supplementary Material

NR-015-D3NR01581E-s001
