# Supplementary material for: Crystal phase engineering of silicene by Sn-modified Ag(111)
Source: Nanoscale. 2023 May 2;15(26):11005–12. doi: 10.1039/d3nr01581e (PMC10324326; doi:10.1039/d3nr01581e)
Supplement: NR-015-D3NR01581E-s001 [file NR-015-D3NR01581E-s001.pdf]

Electronic Supplementary Material (ESI) for Nanoscale.
This journal is © The Royal Society of Chemistry 2023

Supplementary Information for:

Crystal Phase Engineering of Silicene by Sn-modified Ag(111)

Simona Achilli,[a‡] Daya Sagar Dhungana,[b‡] Federico Orlando,[a] Carlo Grazianetti,[b] Christian Martella,[b] Alessandro Molle,[*b] and Guido Fratesi[*a]

S1 LEED of Ag_2Sn on Ag(111)

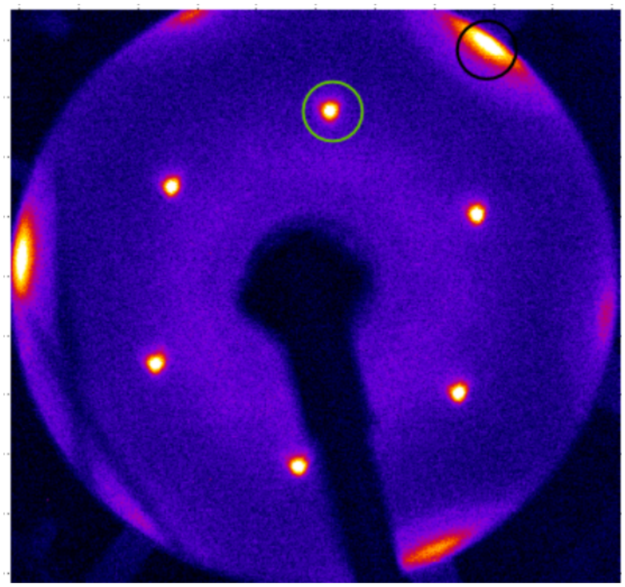

Fig. S1 LEED patterns of Ag_2Sn-Ag(111) surface taken at incident LEED energy of 30 eV. The black circle marks the Ag(111)-1×1 spot, the green one is the first order Ag_2Sn $\sqrt{3}\times\sqrt{3}R30^\circ$ spot.

S2 LEED: Silicene on Ag_2Sn with varying thickness

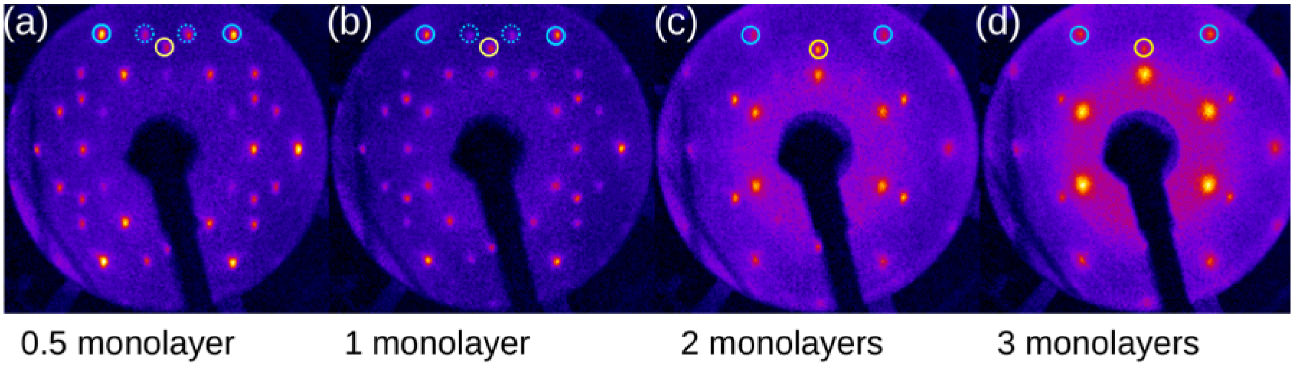

Fig. S2 LEED patterns seen on Ag_2Sn-Ag(111) surface when Si thickness is increased from left to right. All LEED images are taken at 30 eV. The cyan solid circle represents integer order Si spot (4/3 with respect to Ag(111)-1×1), the yellow circle represents Sn first order $\sqrt{3}R30^\circ$ (with respect to Ag(111)-1×1) spot.

[a] ETSF and Physics Department "Aldo Pontremoli", University of Milan, via Celoria 16, Milano I-20133, Italy. E-mail: guido.fratesi@unimi.it
[b] CNR-IMM Agrate Brianza Unit, via C. Olivetti 2, Agrate Brianza I-20864, Italy, E-mail: alessandro.molle@mdm.imm.cnr.it

S3 LEED: Silicene on Sn with varying thickness at submonolayer regime

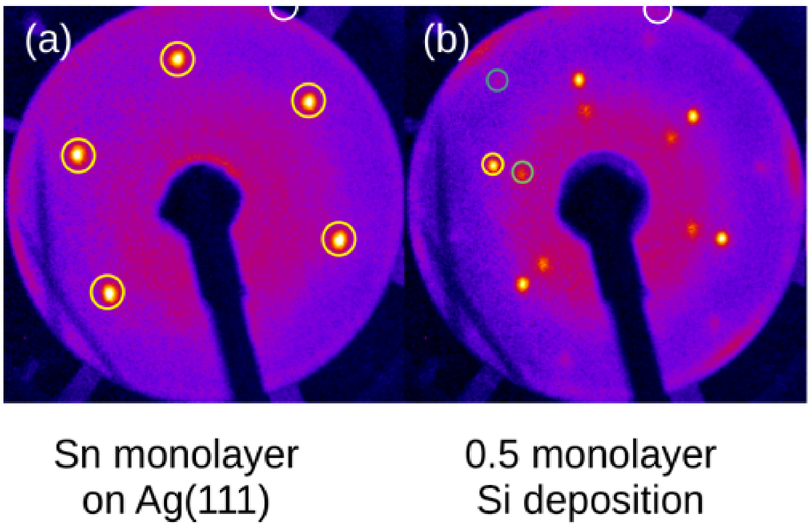

Fig. S3 (a) Sn-Ag(111) template with monolayer Sn on Ag(111). (b) 0.5 monolayer Si deposition on (a). Both images are taken at incident LEED energy 33 eV. Yellow circles are first order $\sqrt{3}R30^{\circ}$ (with respect to Ag(111)-1×1, white circle on the edge) spots, whereas dark and bright green are respectively integer (4/3 with respect to Ag(111)-1×1) and $\sqrt{3}R30^{\circ}$ Si spots ($4/\sqrt{3}R30^{\circ}$ with respect to Ag(111)-1×1).

S4 LEED: Mapping of 4×4 spots

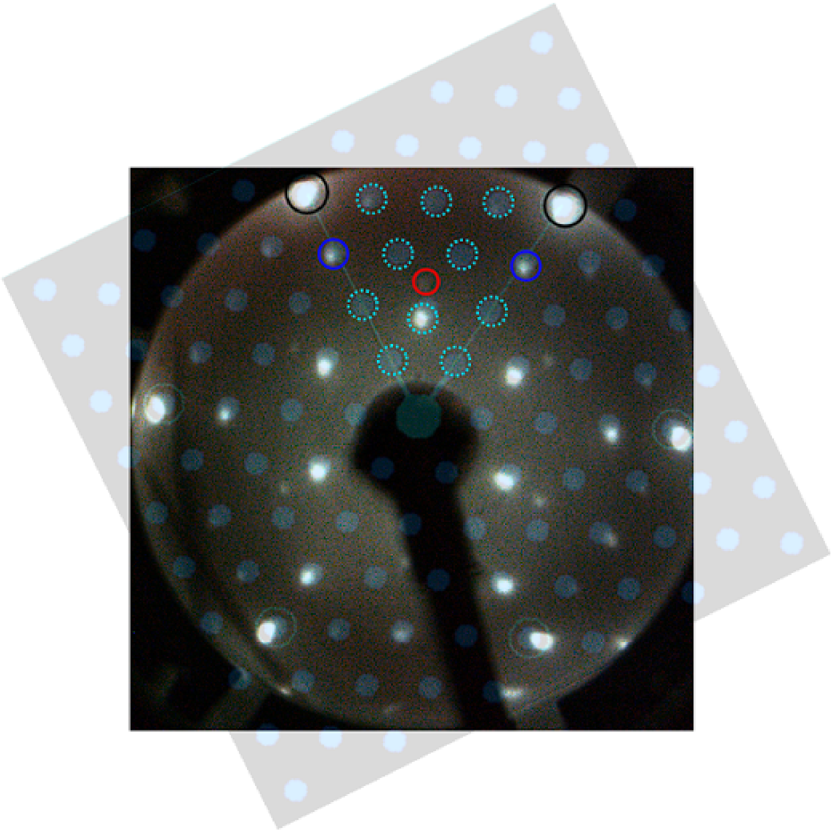

Fig. S4 Mapping of silicene 4×4 single-phase pattern. The LEED image corresponds to the sample reported in Fig. 1e of the main text, here measured at 45 eV. The overlapped pattern was generated with LEEDPat42. The black, blue and red solid circles are: Ag(111)-1×1, the integer spot of the 4×4 phase ($4/3\times4/3$ with respect to the black circle) and $\sqrt{3}\times\sqrt{3}R30^{\circ}$ Sn spots (with respect to the black circle). The dotted cyan patterns are minor spots (shown in one section only) that complete the pattern of the 4×4 reconstruction.

S5 LEED: Mapping of $\sqrt{3}\times\sqrt{3}R30^\circ$ Sn spots

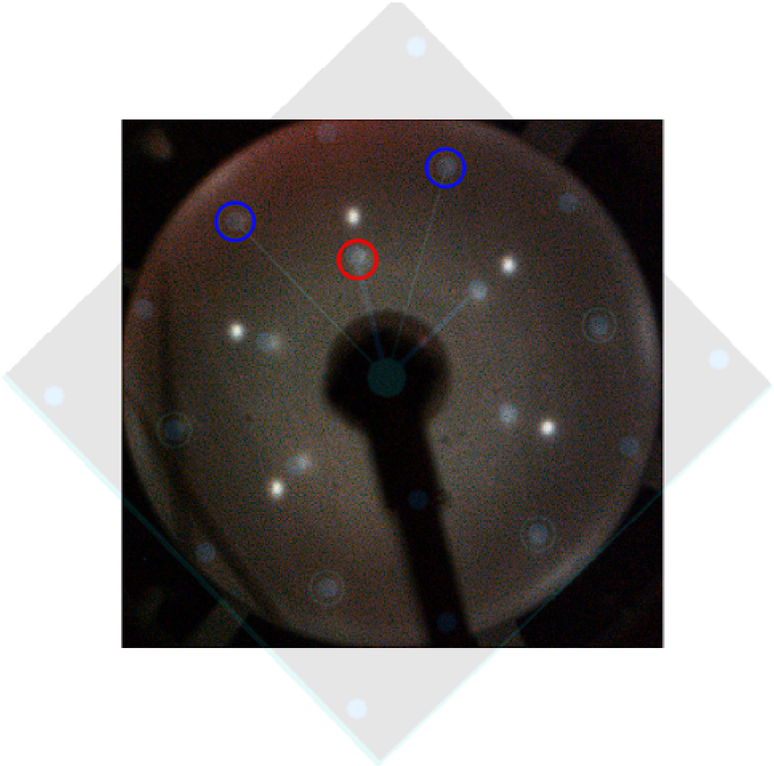

Fig. S5 Mapping of silicene $\sqrt{3}\times\sqrt{3}R30^\circ$. The LEED image corresponds to Fig. 1h of the main text at 30 eV. The overlapped $\sqrt{3}\times\sqrt{3}R30^\circ$ pattern was generated with LEEDPat42. The blue circle is $4/3\times4/3$ with respect to Ag(111)-1×1 and the red is silicene-$\sqrt{3}\times\sqrt{3}R30^\circ$ with respect to the blue one.

S6 LEED: Silicene on Ag(111) grown at different temperatures

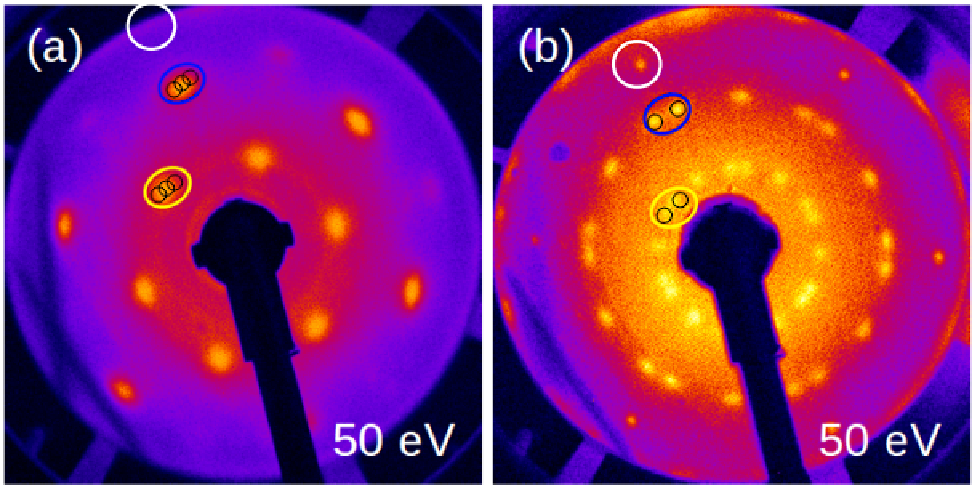

Fig. S6 Multilayer silicene (≈ 3 ML) grown directly on Ag(111) varying in terms of growth temperature as (a) 200 °C (b) 250 °C. Blue ellipses are the integer order Si spots ($4/3\times4/3$ with respect to Ag(111)-1×1 (solid white circles; represented only once). Similarly, yellow ellipses are Si-$\sqrt{3}\times\sqrt{3}R30^\circ$ spots with respect to the blue circle. The small solid black circles, 3 in (a) and (2) in (b), decompose the elongated ellipse into multiple texture. At low temperature (a) the spot starts to elongate, whereas at high temperature (b) the features get completely separated with disappearance of the central $4/3\times4/3$ one.

S7 LEED: Silicene on Sn-engineered interfaces patterns

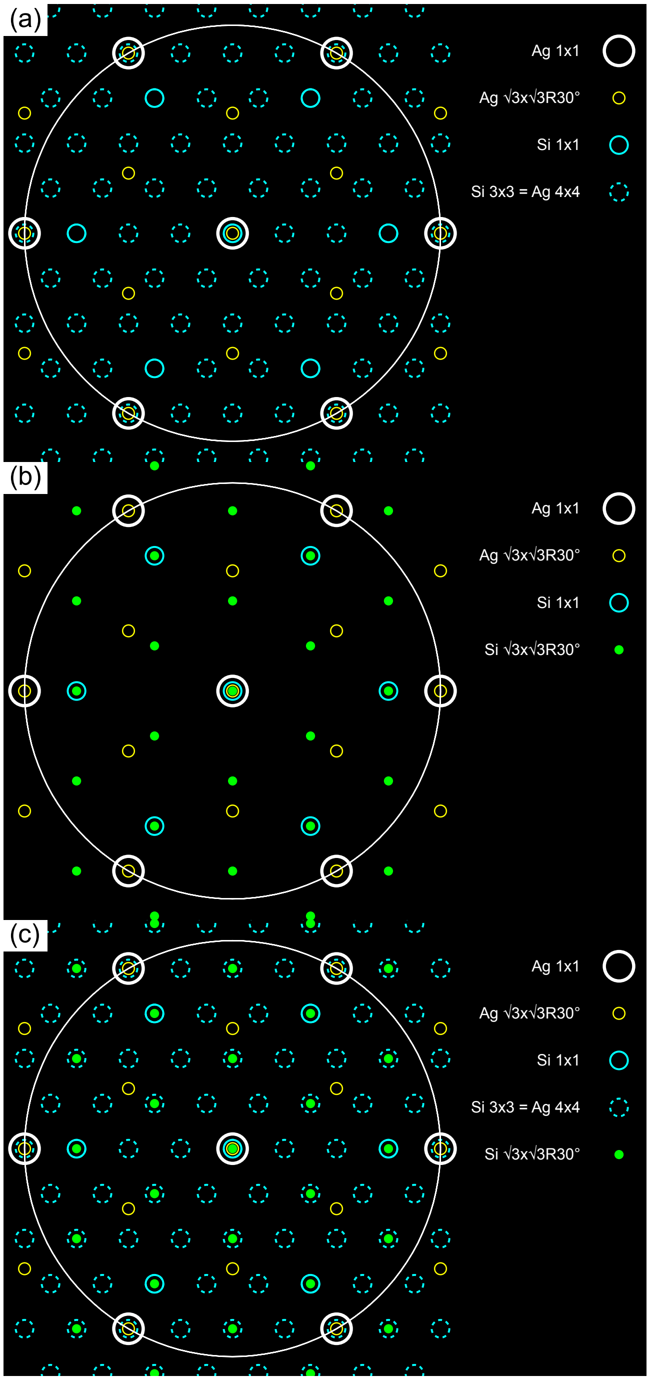

Fig. S7 LEED pattern simulations elaborated for the Ag-4×4/Si-3×3 and the $\sqrt{3}\times\sqrt{3}R30^{\circ}$ phases. Large white circles report the reciprocal lattice of the clean Ag(111) surface (1×1); its $\sqrt{3}\times\sqrt{3}R30^{\circ}$ reconstruction (due to Sn) is shown as yellow circles. Solid cyan circles are the 1×1 spots of silicene; its 3×3 reconstruction, coinciding with a 4×4 reconstruction of Ag(111) (corresponding to the 4×4 phase), is shown with dashed cyan circles, while its $\sqrt{3}\times\sqrt{3}R30^{\circ}$ is marked by green spots. The large circle passing through Ag-1×1 spots approximately corresponds to the area visible in the LEED patterns at 30 eV. Panel (a): pattern as we observe for 0.5 and 1.0 ML of Si on SnAg2-Ag(111), see e.g. Fig. S2. Panel (b): pattern as we observe for multilayer Si on both Sn-engineered interfaces (Fig. 1f and Fig. 1i), as well as for mono- and submonolayer of Si on Sn-Ag(111) (Fig. 1h, Fig. S3). These identify via the green spots a $\sqrt{3}\times\sqrt{3}R30^{\circ}$ reconstruction of a silicene layer that is well aligned with surface azimuths. Panel (c): superposition of patterns shown in panels (a-b); notice that the Si-$\sqrt{3}\times\sqrt{3}R30^{\circ}$ spots (filled green circles) are a subset of Si-3×3 spots (dashed cyan circles), and that Si-1×1 spots (Ag-$4/3\times4/3$ spots) are expected and observed for both 4×4 and $\sqrt{3}\times\sqrt{3}$ phases, whereas they are absent in the $\sqrt{13}\times\sqrt{13}$ and $2\sqrt{3}\times2\sqrt{3}$ silicene phases having different orientations.

S8 Auger Electron Spectroscopy

Fig. S8 presents the AES analysis carried out on mono- and multilayer silicene grown on the aforementioned Ag_2Sn-Ag(111) template. We refer to our previous work for the results on Ag(111) and Sn-Ag(111).[23] The black and red curves in Fig. S8(a) were obtained from the bare Ag(111) and Ag_2Sn-Ag(111) surfaces, respectively. In the inset, two main Sn peaks at 430 and 437 eV can be noticed (red spectrum obtained with higher modulation as tin is less sensitive) without attenuating the main silver peak at 354 eV. This evidence, combined with the LEED pattern showing $\sqrt{3} \times \sqrt{3}R30^{\circ}$ periodicity, confirms incomplete wetting of tin on Ag(111) and therefore the Ag_2Sn-Ag(111) surface. Fig. S8 (b) and (c) respectively focus on the silicon peak at 92 eV and the silver main peak at 354 eV following silicon deposition on Ag_2Sn-Ag(111) template. Green and blue spectra were obtained with silicon thickness of 1 and 6 ML, respectively. In Fig. S8 (b) we notice that the silicon peak increases nearly 3 times with increasing silicon thickness from 1 (green) to 6 ML (blue). In parallel, the main Ag peak attenuates from 75% (green) to almost 30% (blue) intensity with respect to the original silver peak before silicon deposition. The AES monitoring is consistent with the silicon overgrowth with thickness, thus ruling out massive silicon redistribution phenomena like intercalation, as also previously found for the Sn-Ag(111) substrate.[23]

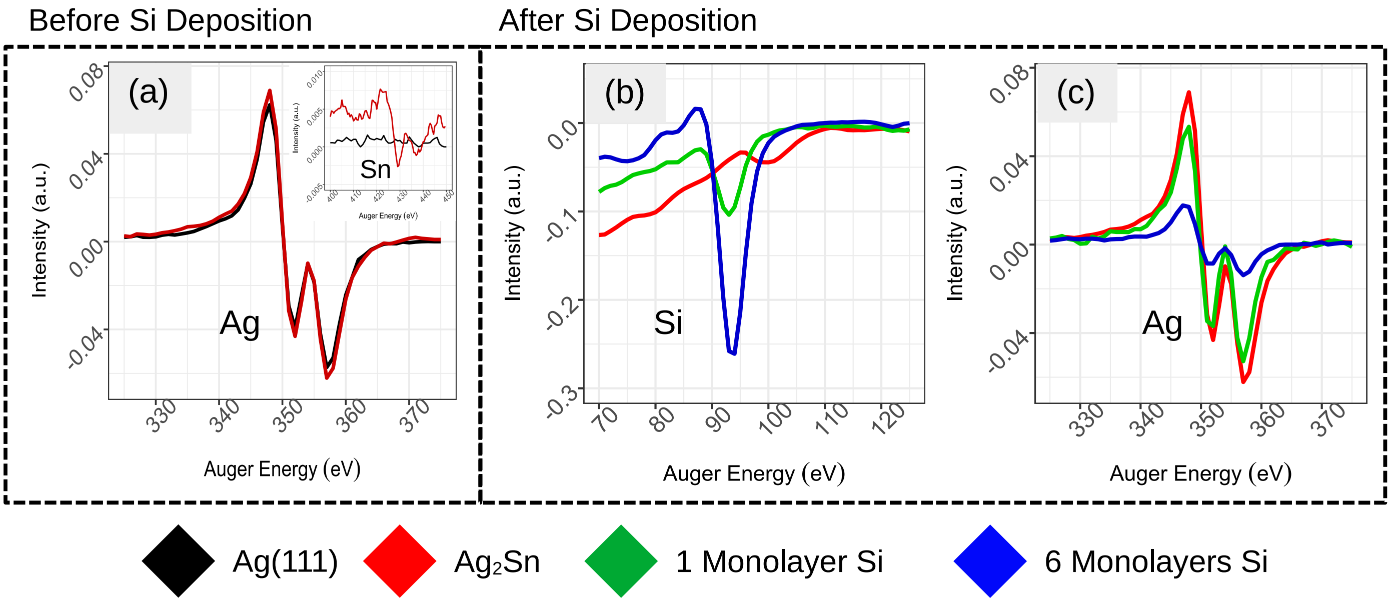

Fig. S8 Auger spectra obtained on various surfaces as labelled with different colors in the legend. (a) Ag_2Sn-Ag(111) surface before silicon deposition. The inset focuses on tin (acquired with higher modulation). (b) and (c) after growth, focusing on silicon main peak at 92 eV and silver main peak at 354 eV. Green and blue spectra refer to silicon thickness of 1 and 6 ML, respectively.

S9 Silicene geometries

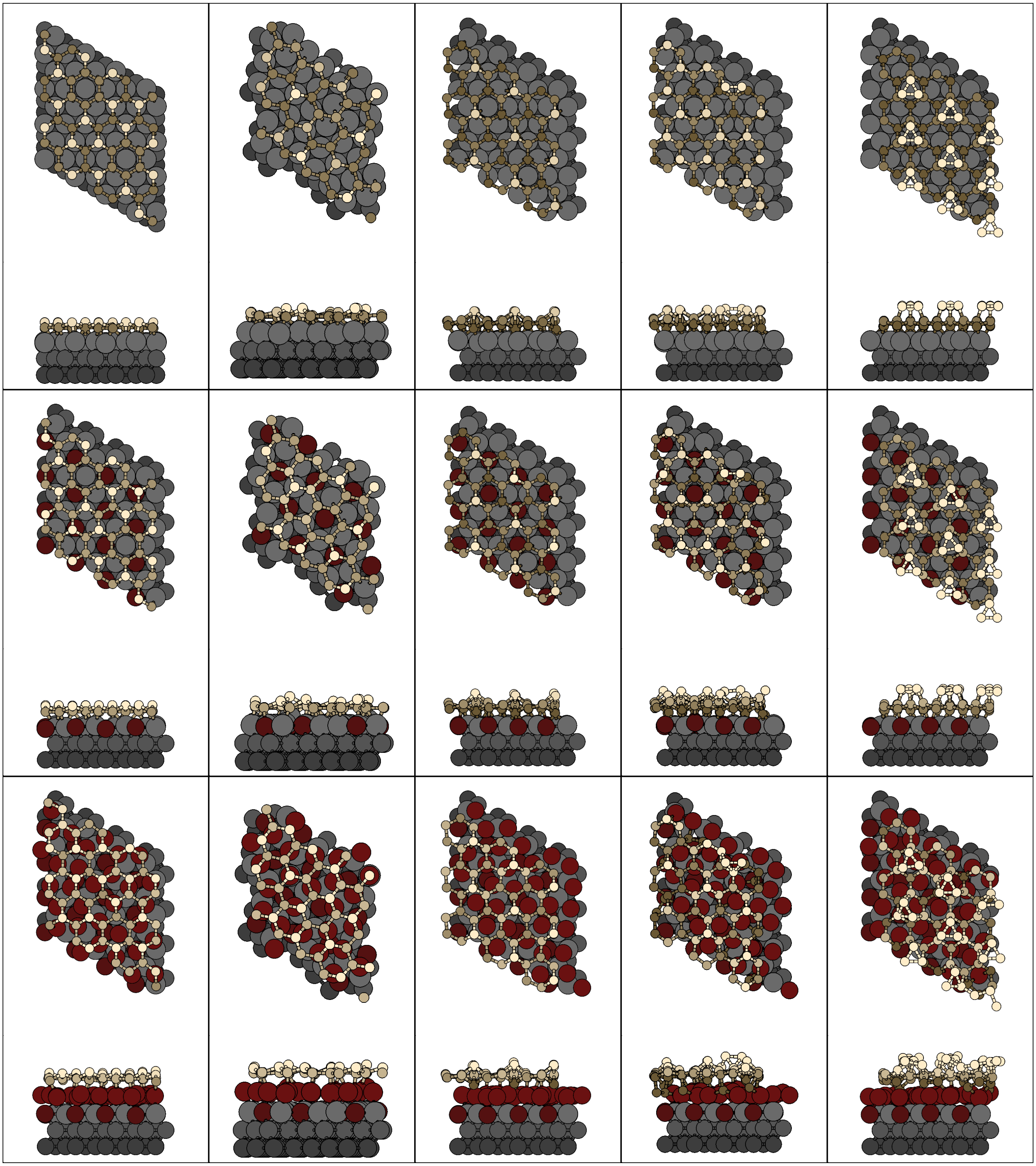

Fig. S9 Top and side views of model structures for all phases investigated here. From left to right, 4×4, $\sqrt{13}\times\sqrt{13}$, TDS, HDS and SiT. From top to bottom, Ag(111) substrate, Ag_2Sn-Ag(111) and Sn-Ag(111). Si atoms are represented as yellow spheres, Sn as red and Ag as gray ones; darker colors are used for deeper atoms.

	4×4		$\sqrt{13}\times\sqrt{13}$		TDS		HDS		SiT	
	z (Å)	b (Å)	z (Å)	b (Å)	z (Å)	b (Å)	z (Å)	b (Å)	z (Å)	b (Å)
Ag(111)										
Si	2.36	0.88	2.35	1.14	2.79	2.70	3.09	2.88	3.42	3.07
Ag_2Sn-Ag(111)										
Si	2.52	1.00	2.51	1.54	3.00	3.00	3.27	3.70	3.60	3.27
Sn-alloy	-0.20	0.26	-0.22	0.24	-0.10	0.39	0.02	0.70	-0.10	0.20
Sn-Ag(111)										
Si	5.44	1.56	5.49	1.20	5.67	3.39	5.63	5.44	6.16	4.91
Stanene	2.65	0.54	2.68	0.76	2.66	0.61	2.71	1.04	2.64	0.80
Sn-alloy	-0.07	0.25	0.00	0.26	-0.06	0.25	-0.01	0.63	-0.01	0.22

Table S1 Average height (z) and buckling (b, defined as $b = z_{max} - z_{min}$) for Si and Sn atoms of the models considered. Heights are measured with respect to the average height of the Ag atoms in the first surface layer.

S10 DFT electronic properties

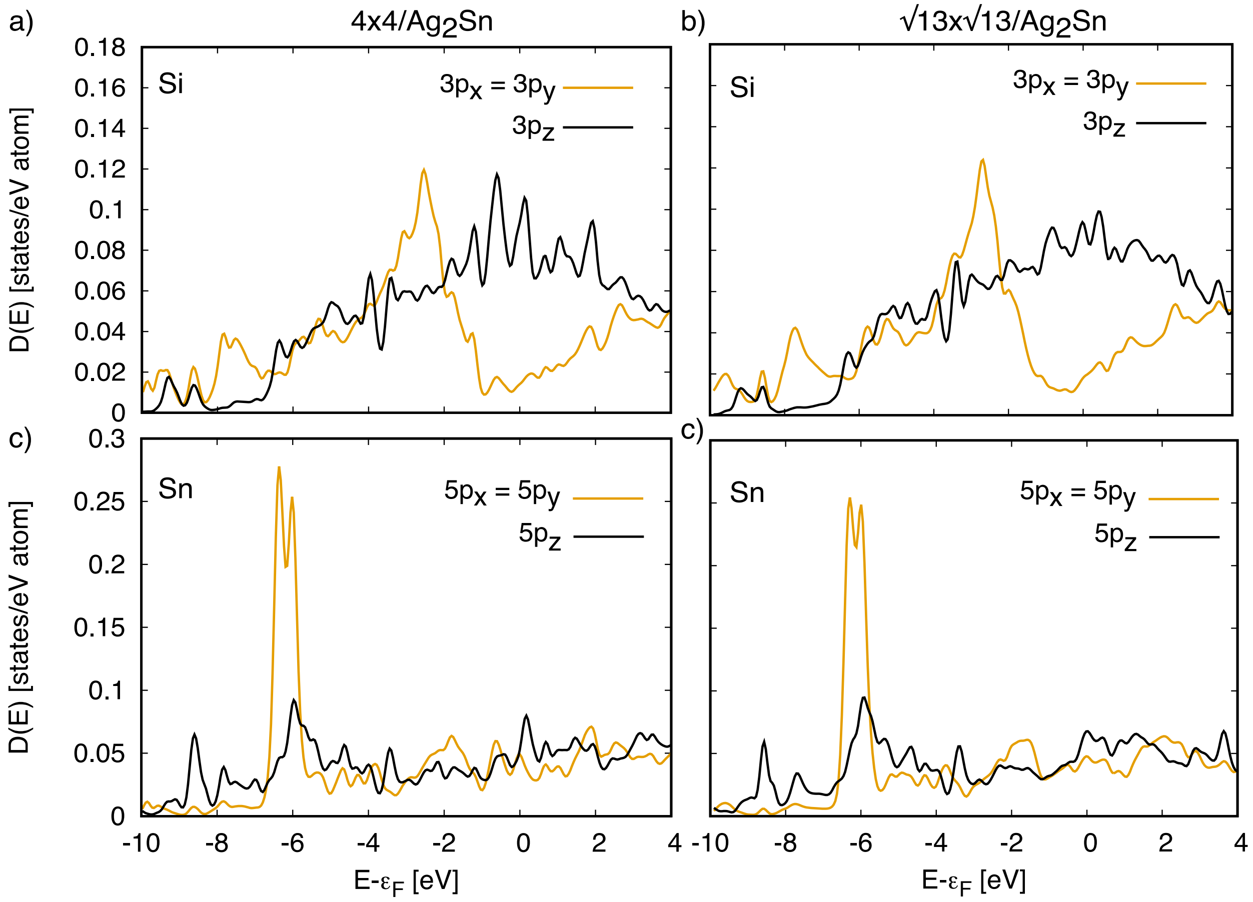

Fig. S10 Density of states projected on p_x, p_y, p_z components for Si (a, b) and Sn (c, d) in the 4×4 and $\sqrt{13}\times\sqrt{13}R13.9°$ on Ag_2Sn.

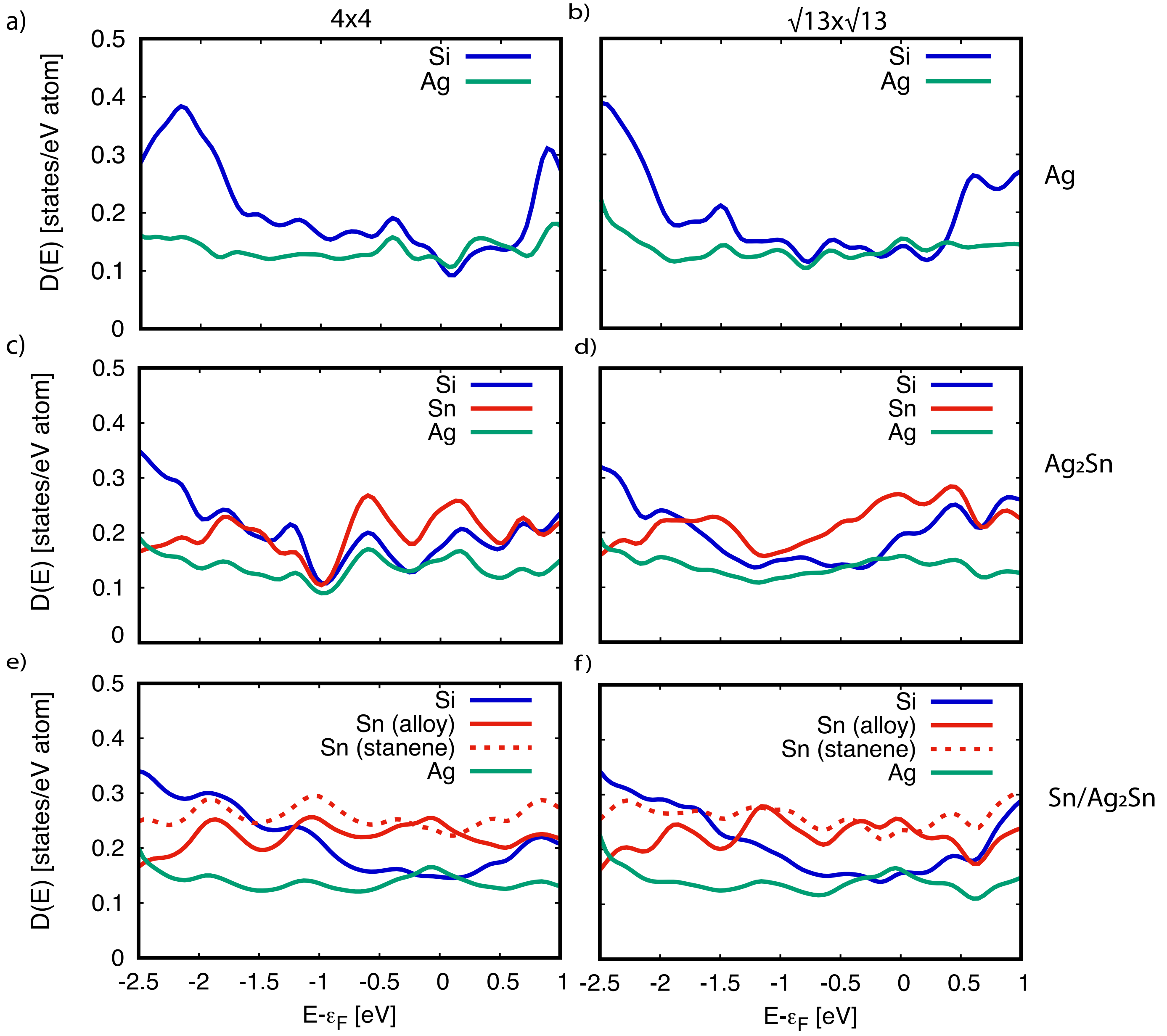

Fig. S11 Density of states projected on Si, Ag and Sn for the 4×4 and $\sqrt{13}\times\sqrt{13}R13.9^{\circ}$ on Ag (a, d), Ag_2Sn (b, e) and Sn/Ag_2Sn (c, f).

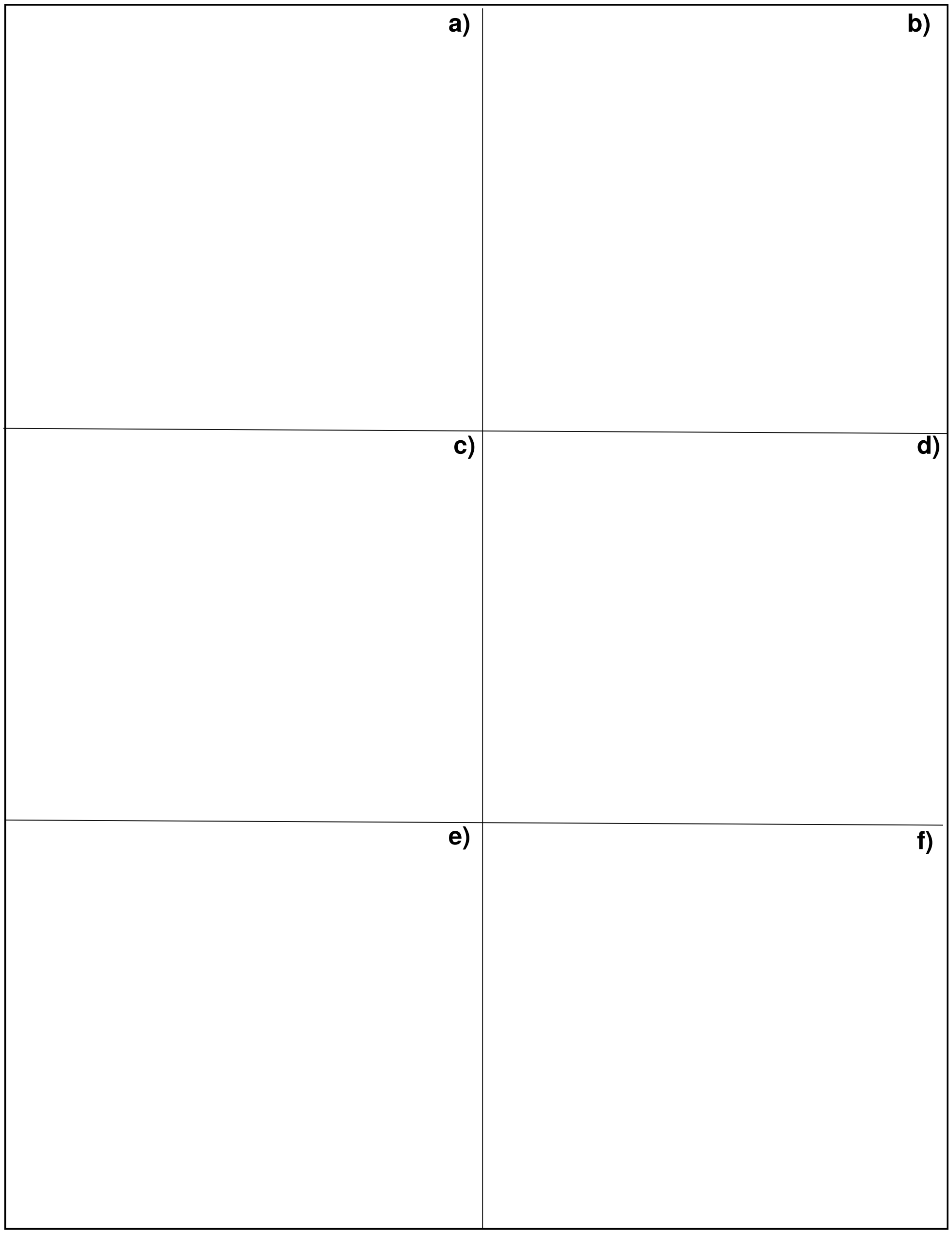

Fig. S12 Charge transfer for the less stable phases. Color code for Si partial as in Fig. 7b.

Looking at the charge transfer for the less stable phases (Fig. S12) there are some differences to note. The $\sqrt{3} \times \sqrt{3}$ configurations on Ag and Ag_2Sn-Ag(111) are characterized by positive charge transfer to Si, at odds with what reported in Fig. 7a for TDS and HDS, due to the absence of a Sn buffer layer, accordingly to what happens for the other phases on the same substrate. The general trend of a smaller interaction between Si and the substrate when Ag is covered by an alloy overlayer is confirmed, although a sizeable charge redistribution in silicene is found. In particular, in SiT/Ag_2Sn the net charge transfer to Si is null (in Fig. S12) whereas a large charge separation in the silicene sublayers with respect to the freestanding system is detected, that may be a hint of an overall instability of the overlayer. A similar effect holds for HDS/Ag_2Sn, as well.

Concerning the 4×4 and $\sqrt{13} \times \sqrt{13}$ on Sn-Ag(111), we find quite a small charge depletion for Si atoms in the former, including the partial charge on the sublayers, whereas for the latter the small charge transfer from Si is accompanied by a huge charge redistribution in the sublayers. On the basis of the previous analysis, we may suppose that the higher energy of these phases on Sn is related to the absence of interaction with the substrate for the 4×4, which is combined with an induced charge-instability in the $\sqrt{13} \times \sqrt{13}$ on the same substrate.
